# An individual patient-data meta-analysis of metronomic oral vinorelbine in metastatic non-small cell lung cancer

**DOI:** 10.1371/journal.pone.0220988

**Published:** 2019-08-20

**Authors:** Jean-Louis Pujol, Amandine Coffy, Andrea Camerini, Athanasios Kotsakis, Manlio Mencoboni, Milena Gusella, Felice Pasini, Aldo Pezzuto, Giuseppe Luigi Banna, Cemil Bilir, Epaminontas Samantas, Fabrice Barlesi, Benoît Roch, Aude Guillou, Jean-Pierre Daurès

**Affiliations:** 1 Department of Thoracic Oncology, Montpellier Regional University Hospital, Montpellier, France; 2 Laboratory of Biostatistics and Epidemiology, University Institute for Clinical Research, Montpellier, France; 3 Medical Oncology, Ospedale Villa Scassi, Genoa, Italy; 4 Hellenic Oncology Research Group (HORG), Athens, Greece; 5 Medical Oncology, Versilia Hospital—ASL Toscana Nord-Ovest, Lido di Camaiore, LU, Italy; 6 AULSS5 Polesana, UOC Oncologia, Ospedale Santa Maria della Misericordia, Rovigo, Italy; 7 Oncology Service, Casa di Cura Pederzoli, Peschiera del Garda (VR), Italy; 8 Thoracic and Cardiovascular Science Department S. Andrea Hospital, Sapienza University, via Grottarossa, Rome; 9 Division of Medical Oncology, Cannizzaro Hospital, Catania, Italy; 10 Department of Medical Oncology, Faculty of Medicine, Sakarya University, Sakarya, Turkey; 11 Hellenic Cooperative Oncology Group (HeCOG), Athens, Greece; 12 Assistance Publique Hôpitaux de Marseille, Multidisciplinary Oncology and Therapeutic Innovations Department, Aix Marseille University, Centre d’Investigation Clinique, Marseille, France; 13 Medical Direction Department, Pierre Fabre Oncology, place Abel Gance Boulogne-Billancourt, France; European Institute of Oncology, ITALY

## Abstract

**Introduction:**

Several non-comparative phase II studies have evaluated metronomic oral vinorelbine (MOV) in metastatic non-small cell lung cancer (NSCLC) but the small size of each study limits their conclusions.

**Purpose:**

To perform an individual patient-data metaanalysis of studies evaluating MOV in metastatic NSCLC in order to measure survival and safety of treatment with this regimen.

**Methods:**

Studies were selected if (1) administration of oral vinorelbine thrice a week; (2) fixed daily dose comprised between 30 and 50 mg, and; (3) being published before October 4^th^ 2018. Database encompassed 8 variables characterizing disease and demography, 3 informing therapy, and 12 describing survival and toxicity.

**Results:**

Nine studies encompassing 418 patients fulfilled the selection criteria, 80% of them having frailty characteristics. Median overall survival (OS) was 8.7 months (95%CI: 7.6–9.5). OSrates at 6 months, one year and at two years after starting vinorelbine were 64%, 30.3% and 8.9%, respectively. In the Cox model, Eastern Cooperative Oncology Group (ECOG) performance status (PS) = 2, and anemia of any grade were significant determinants of shorter OS. Median progression-free survival(PFS) was 4.2 months (95%CI: 3.9–5). At 6 months and at one-year, PFS rates were 35% and 11.9% respectively. In the Cox model stratified for the variable “study”, PS = 2and stage IV were significant determinants of shorter PFS. No toxicity was reported for 40% of patients, and 66 (15.8%) patients experienced a grade 3–4 toxicity. The most frequent toxicity was anemia of any grade (35.8%) that was higher with the 50 mg dosage.

**Conclusion:**

MOV is an active and well-tolerated chemotherapy in metastatic NSCLC and is a manageable therapy in frail patients.

## Introduction

Lung cancer is the leading cause of cancer mortality among women and men in most developed countries [1].Patients who are diagnosed with metastatic non-small cell lung cancer (NSCLC) are candidates for systemic treatments [2,3]. When activating mutations, such as EML4- ALK rearrangement or EGFR sensitizing mutations, are detected, patients might receive targeted therapy [4], whereas for those without a druggable mutation, but with a tumor expressing programmed death ligand 1 (PD-L1) on more than 50% cancer cells, immunotherapy using immune check point inhibitors is recommended [5].Recently, several studies investigated the combination of immune checkpoint inhibitors and chemotherapy and suggest an outcome improvement over chemotherapy; however, the approval of these approaches is still limited to some countries. Others patients are offered chemotherapy as a palliative treatment, which is also the recommended treatment for those who have progressed after first line immunotherapy or targeted therapy [3]. Most of the patients with good PS and normal end organ functions are eligible for platinum-based doublet chemotherapy combining a platinum compound and a third-generation drug (vinorelbine, docetaxel, pemetrexed, paclitaxel, gemcitabine) [6].

Although platinum-based doublets are the recommended regimens in metastatic NSCLC, even in the elderly [7,8], a significant proportion of NSCLC patients are unfit for this treatment due to functional impairment, unfavorable PS, high comorbidity index, or a combination of these variables. For these patients, single-drug chemotherapy might be offered and consists of a third generation cytotoxic agent given alone [9]. Hence, some drugs such as docetaxel or pemetrexed are approved for subsequent therapeutic lines once the disease became platinum-refractory [6]. As a large number of NSCLC patients requires a treatment option other than doublet chemotherapy at one point during the course of the disease, searching for active single-drug chemotherapy regimens is warranted.

Metronomic chemotherapy has been defined as the frequent administration of chemotherapeutic drugs at doses which are significantly lower than the maximum tolerated dose, and which are delivered without prolonged drug-free breaks [10]. Pre-clinical experiments have suggested that metronomic chemotherapy allows a direct targeting of the tumor vasculature, the immune system and the cancer cells [11,12]. Vinorelbine, a semi-synthetic vinca-alkaloid, has an oral formulation and a good safety profile [13]. It is therefore a good candidate for metronomic chemotherapy in NSCLC [10]. Several phase II studies have evaluated metronomic oral vinorelbine in metastatic NSCLC In these studies, vinorelbine was delivered as a single agent, thrice a week, in populations mainly consisting of patients unfit for platinum-based doublet chemotherapy and of patients whose disease was refractory to first-line, or subsequent-lines of chemotherapy. Overall, these studies suggested an activity of metronomic oral vinorelbine and a good safety profile. However, there are no randomized studies comparing metronomic oral vinorelbine with standard treatments and the small size of each study limits their conclusions.

In this article, we report the results of an individual patient-data metaanalysis of all studies that reported metronomic oral vinorelbine in metastatic NSCLC and that used a thrice-weekly treatment schedule. Individual data were obtained from principal investigators. The primary endpoint was overall survival (OS). The secondary objectives were progression-free survival (PFS) and frequency of National Cancer Institute Common Terminology Criteria for Adverse Events, (NCI-CTCAE overall and grade 3–4) toxicities with particular attention to specific vinorelbine toxicity.

## Methods

### Selection of studies

A comprehensive search was performed with the following MeSH terms: vinorelbine; administration, metronomic; administration, oral; and non-small cell lung cancer. We searched PubMed, MEDLINE, and EMBASE for controlled clinical studies eligible for this metaanalysis published before or on October 4^th^2018. The pharmaceutical company owning the oral vinorelbine license (Pierre Fabre Oncology, Boulogne, France) was asked to share their knowledge about putative unpublished and ongoing studies. At cutoff, ClinicalTrials.gov had registered no ongoing clinical trials using oral nivorelbine in metronomic administration as single-drug therapy for NSCLC; in addition, all relevant clinical trials registered by ClinicalTrials.gov that were declared as ‘closed to enrollment” had been published in the English medical literature.

### Study eligibility

To be eligible, studies need to have prospectively accrued patients with advanced or metastatic NSCLC regardless of patient age. The enrolled populations need to have the following characteristics: (1) histologically or cytologically proven non-small cell cancer, (2) an advanced or metastatic stage, according to the criteria of the 7^th^ or 8^th^Union Internationale contre le Cancer-American Joint committee TNM classification(Stage III and IV definition takes into account the work up described in the eligibility criteria of each individual study); (3) Eastern Cooperative Oncology Group (ECOG) performance index (PS)at inclusion of 0–2; (4) measurable disease with tumor assessment at preplanned intervals; and (5) no symptomatic brain metastasis (patients with asymptomatic brain metastases were eligible if a treatment was undertaken for the control of brain metastases).

In addition, the chemotherapy treatment of each study need to comply with the following procedure: (1) administration of oral vinorelbine using the metronomic regimen thrice a week (Mondays, Wednesdays, Fridays) and (2) vinorelbine administration at a fixed daily dose comprised between 30 and 50 mg,and (3) information patient-by-patient of the systemic treatment sequence (chemonaive-patient, or second line, or subsequent line). Studies in which administration was not done on Mondays, Wednesdays and Fridays, but used other schedules such as daily administration, or administration every other week were excluded. Nevertheless, studies considering administration every other day were selected, insofar as this schedule approximated the thrice weekly administration. In addition, studies that used a daily dosage of vinorelbine lower than 30 mg were not included in the metaanalysis regardless of the method of administration.

Corresponding authors of eligible studies were individually contacted by mail and invited to provide individual patient-data. Two files were attached to this invitation: (1) the study protocol including, aim of the metaanalysis, endpoints, process, statistical plan, list of needed variables for each patient and publication rules; (2) the excel file to be filled out by each study manager. A database was created that encompassed 8 variables characterizing disease and demography, 3 informing therapy, and 12 describing survival and toxicity.

### Patient eligibility

Individual patients were included if data for each patient encompassed (1) evidence of histological or cytological proven NSCLC, (2) valid cancer stage grouping demonstrating either a locally advanced or a metastatic disease; (3) ECOG PS < = 2; (4) valid information regarding the place of vinorelbine treatment in the sequence of disease management (first line, second line, versus subsequent line); (5) no symptomatic brain metastases; (6) treatment with thrice weekly schedule; (7) valid information regarding the administered dosage of oral vinorelbine.

### Outcomes

The primary endpoint was OS inthe intention-to-treat population. Overall survival was defined as time from the first day of metronomic oral vinorelbine to death from any cause. Secondary endpoints were (a) PFS, defined as time from the first day of metronomic oral vinorelbine to either disease progression or death from any cause, whichever occurred first and (b) percentage of patients affected by vinorelbine specific toxicities (neutrophil counts, platelet counts, hemoglobin titration and emesis, evaluated according to the NCI-CTC vs 4.0). In two studies (Bilir et al. 2017and D’Ascanio M. et al. 2018), survival data were incomplete inasmuch was some patients were known as experiencing progression with a censored data for OS, a long period before the study cutoff. For these two studies with putative informative censorship, a penalizing survival analysis was applied so that, the OS was defined as the time from the first day of metronomic oral vinorelbine to death from any cause or date of last contact. The Response Evaluation Criteria in Solid Tumor were not universally used across the different studies. Therefore, the response rate was not considered as an endpoint in this individual-patient data metaanalysis.

### Statistics

Descriptive analyses were done study-by-study. Thereafter, there were done in the intention-to-treat metaanalysis population. Quantitative variables were described by their mean, and median. The qualitative variables were described by their size, percentage (n,%).

Survival analyses were processed as follows: the cutoff date for the entire population of the metaanalysis was defined as the latest date for which we have an informative point (death or censorship). This date was the point from which the events of all the studies were either deaths or censorship (lost to follow up or administrative censorship of the corresponding study).

The non-parametric Kaplan-Meier estimates were used to calculate probabilities and plot survival curves. Survival distribution of groups defined by the different states of a covariable was compared using the log-rank test. Covariates related to the occurrence of the survival event with a p <0.20 were included in a multivariate COX hazard proportional model. However, the variable “study” was included in the model regardless of the log-rank test in order to search for a “study” effect. The proportional hazard assumption was tested graphically [function LOG (-LOG (S (t))] and, where needed, by a time-dependent Cox model. Where covariates did not meet the proportional hazard assumption, a stratified Cox model was applied. Stepwise, backward and forward variable selections were tested. The variables with an alpha risk of 5% were selected in the final model. In order to estimate the robustness of the results and to test heterogeneity between studies, the model was run again by recalculating after having successively suppressed and reintroduced the studies, one-by-one (jackknife).

## Results

### Studies and population

**Fig 1:**
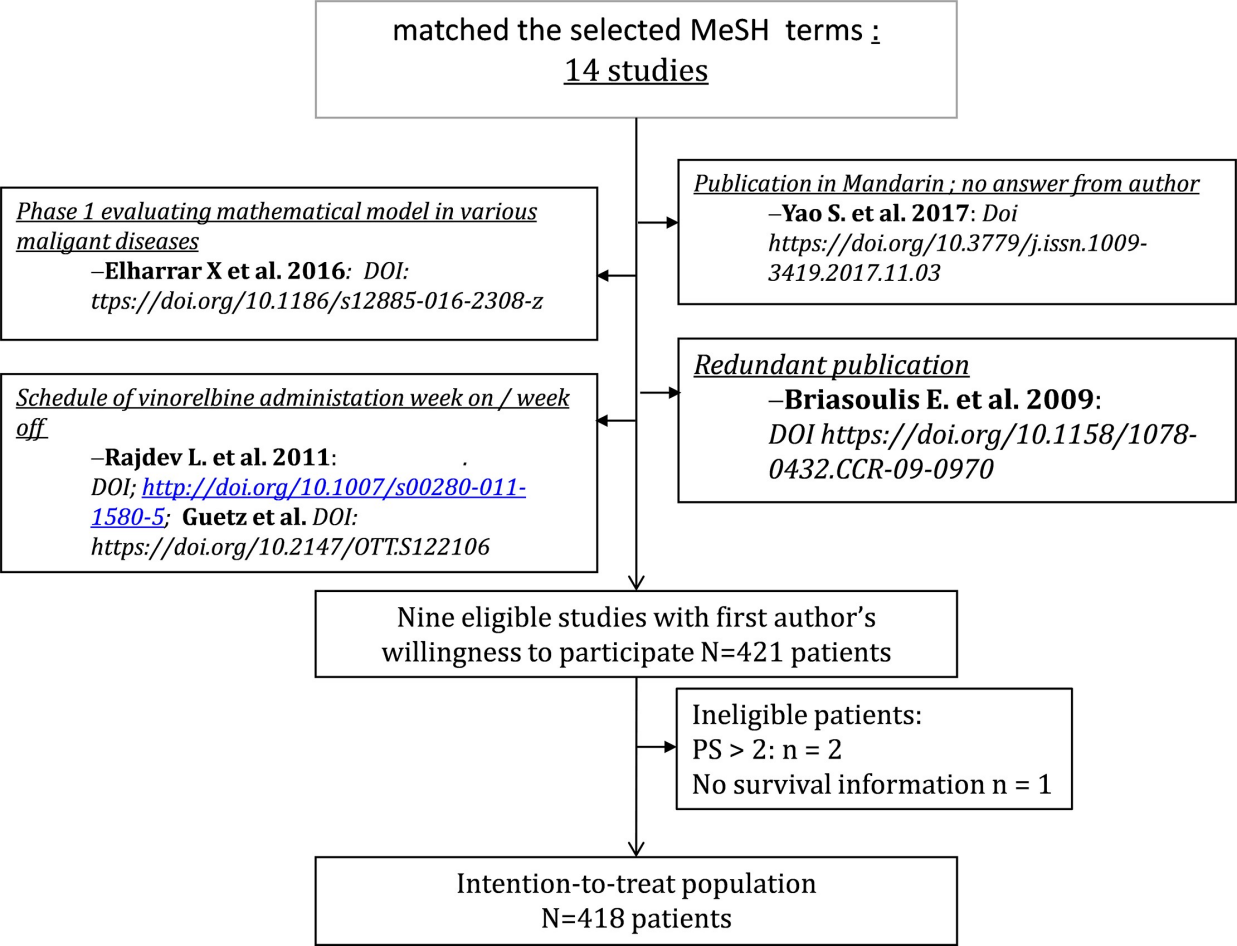
CONSORT diagram of the metaanalysis of metronomic oral vinorelbine in non-small cell lung cancer.

The first step of the selection process identified fourteen studies that matched the selected MeSH terms (Fig 1). After careful analysis of each publication, four studies were found to be ineligible: the study by Elharrar X et al [14] investigated a mathematical model in a phase I study with various malignant diseases (pharmacological evaluation as primary endpoint); two studies (Rajdev L. et al. 2011 and Guetz et al. 2017) [15,16] used a daily administration of oral vinorelbine with a “week-on, week-off“schedule; one study was a redundant publication with fewer accrued patients in the first publication^17^than in the final publication (Briasoulis E. et al. 2009 [17], n = 14; Briasoulis E. et al. 2013, n = 31); finally, one study analyzing 26 patients, and published in mandarin seemed eligible according to the English abstract, albeit retrospective [18]. The authors were contacted in order to test feasibility of including their study in this individual patient-data metaanalysis but could not be reached despite repeated attempts. This study would have contributed to the final metaanalysis at a level of 5% in the case of authors' willingness to participate.

A total of nine studies was selected to be included in the individual patient-data metaanalysis, all with a positive first author’s answer to cooperate. Table 1 describes authorship, year of publication, journal and digital object identifier. The number of patients accrued in each study, according to the original publication, was generally congruent with the number of patients included in each received database (range 9–92). In one study, the number of patients in the database slightly exceeded the published one and in another study, two patients were missing when compared with the published study report. The merged database included 421 patients. After carefully browsing of each line, three patients were excluded from the analysis: two of them had a ECOG PS > 2 and the third had missing data on the primary endpoint (OS). Eight patients incidentally received a 20 mg vinorelbine dosage with a thrice weekly schedule; this was considered as a minor deviation from the eligibility criteria and these 8 patients were included in the dataset. Consequently, the intention-to-treat population for this metaanalysis included 418 patients. Two of the studies (Mencoboni et al; Camerini et al) partially reported safety variables insofar as patients affected by a grade 3–4 toxicity were identified but the specific toxicity was not reported. As a result, the safety dataset of the metaanalysis included 300 patients.

**Table 1:** Selected studies in the metaanalysis on metronomic oral vinorelbine in NSCLC.

Authors	Journal	Title and DOI	Vinorelbine dose (mg/d)	NSLC patients # in publication	NSCLC patients # in database
Briasoulis E. et al. 2013	BMC Cancer	Dose selection trial of metronomic oral vinorelbine monotherapy in patients with metastatic cancer: a hellenic cooperative oncology group clinical translational study https://doi.org/10.1186/1471-2407-13-263	30/40/50	31	31
Camerini A. et al. 2015	BMC Cancer	Metronomic oral vinorelbine as first-line treatment in elderly patients with advanced non-small cell lung cancer: results of a phase II trial (MOVE trial). https://doi.org/10.1186/s12885-015-1354-2	50	43	43
Mencoboni M. et al. 2017	AnticancerRes.	Safety of First-line Chemotherapy with Metronomic Single-agent Oral Vinorelbine in Elderly Patients with NSCLC. https://doi.org/10.21873/anticanres.11679	50	76	76
Bilir et al. 2017	CurrOncol.	Efficacy of metronomic vinorelbine in elderly patients with advanced non-small-cell lung cancer and poor performance status. https://doi.org/10.3747/co.24.3486	30	35	35
Kontopodis E. et al. 2013	J Chemother.	A phase II study of metronomic oral vinorelbine administered in the second line and beyond in non-small cell lung cancer (NSCLC): a phase II study of the Hellenic Oncologic Group	50	46	46
		https://doi.org/10.1179/1973947812Y.0000000050			
Banna GL. et al. 2018	AnticancerRes.	Oral Metronomic Vinorelbine in Advanced Non-small Cell Lung Cancer Patients Unfit for Chemotherapy. https://doi.org/10.21873/anticanres.12647	30	41	41
Barlesi F. et al. 2017	Oncotarget	Mathematical modeling for Phase I cancer trials: A study ofmetronomicvinorelbine for advanced non-small cell lung cancer(NSCLC) and mesothelioma patients. https://doi.org/10.18632/oncotarget.17562	60 mg on Day 1, 30 mg on Day 2 and 60 mg on Day 4	9	9
Pasini F. et al. 2018	Investigational New Drugs	Oral Metronomic Vinorelbine (OMV) in elderly or pretreated patients with advanced non small cell lung cancer: outcome and pharmacokinetics in the real world. https://doi.org/10.1007/s10637-018-0631-8	20/30/50	92	90
D’Ascanio M. et al. 2018	BioMed Research International	Metronomic Chemotherapy with Vinorelbine Produces Clinical Benefit and Low Toxicity in Frail Elderly Patients Affected by Advanced Non-Small Cell Lung Cancer doi: 10.1155/2018/6278403	30/40	44	50

### Description of the population

The median contribution of each study to the metaanalysis was 43 patients (10.3%; range 9 patients [2.5%]– 90 [21.5%], Table 2). Most of the patients were male (331 patients: 79.2%). Mean +/- SD age at the time of accrual was 72,8 +/- 9,1 years and 198 patients were 75 years of age or older (47.4%). Adenocarcinoma was the predominant histology (204 patients; 48.8%) whereas squamous-cell carcinoma or large-cell carcinoma and not otherwise specified NSCLC were diagnosed in 184 patients (44%); sub-histology of NSCLC was a missing data in the remaining 30 patients (7,2%). Most of the patients had a stage IV disease (82.1%). A total of 238 patients (56.9%) had a favorable PS 0–1. In order to better characterize the frailty of the cohort, a frailty score was constructed as the sum of each of the following features: age 75 years or older (+1), administration of vinorelbine as the third line of treatment or subsequent line (+1), PS = 2 (+1), and Charlson comorbidity index = 3 or greater (+1). Missing data for a given item were classified as zero. Eighty percent of the patients presented with 1 to 4 frailty characteristics (Table 2).

**Table 2:** Patients demographics and disease characteristics in the intention-to-treat population of the metaanalysis on metronomic oral vinorelbine in non-small cell lung cancer.

Variables	Status	N	%
Gender	Female	87	20.8
	Male	331	79.2
Age	< 75 years of age	219	52.4
	≥ 75 years of age	198	47.4
	Missing data	1	0.2
Histology	ADE	204	48.8
	Non-ADE	184	44.0
	Un specified NSCLC	30	7.2
Vinorelbine dose	20	8	1.9
	30	166	39.7
	40	53	12.7
	50	182	43.5
	Adaptative dosage	9	2.2
Stage grouping	III	75	17.9
	IV	343	82.1
ECOG Performance Status	0/1	238	56.9
	2	180	43.1
Vinorelbine chemotherapy sequence	First line	200	47.8
	Second line	139	33.3
	Subsequent line	48	11.5
	Missing data	31	7.4
Frailty score #	0	80 (19.1)	19.1
	1	174 (41.5)	41.5
	2	108 (25.8)	25.8
	3	53 (12.7)	12.7
	4	3 (0.7)	0.7

Abbreviations used: NSCLC: non-small cell lung cancer; ADE: adenocarcinoma; ECOG: Eastern Cooperative Oncology Group; #The frailty score was constructed as the sum of each of the following features: age 75 years or older, administration of vinorelbine as third line of treatment or subsequent line, ECOG performance status = 2, and Charlson comorbidity index = 3 or greater.

The most frequently administered daily vinorelbine dosages were 30 and 50 mg. Two hundred patients received metronomic oral vinorelbine in the first line (47.8%).

### Overall survival

Median follow-up (range) for OS analysis was 7.8 months (0.4–49.0). There were 338 events reported by the investigators in the nine studies (80.9% of the intention-to-treat population). After penalization of two studies (see method section), 371 events (deaths) were considered in the OS analysis (88.8%). Consequently, 11.2% of the patients were censored at the time of analysis.

**Fig 2:**
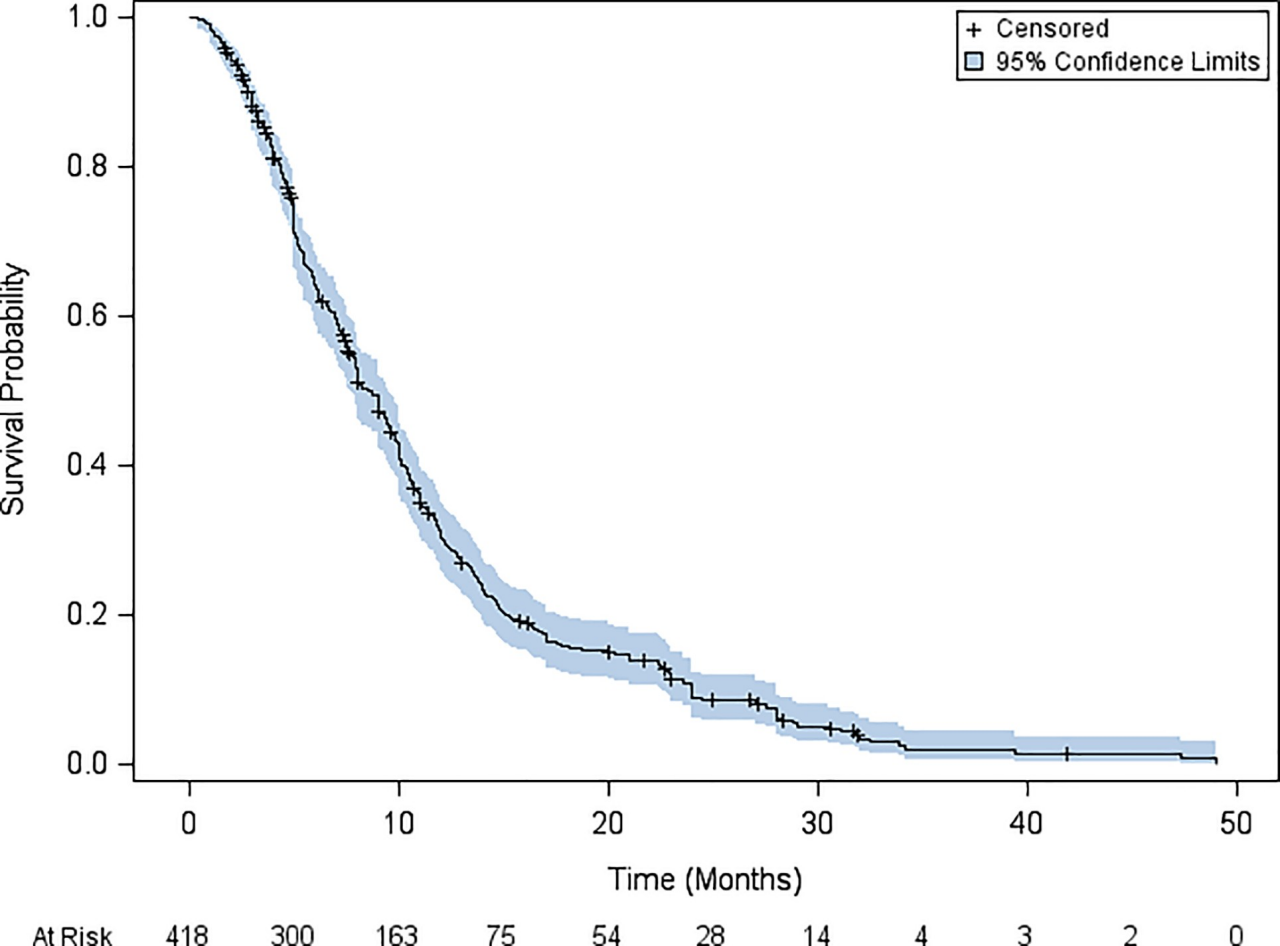
Kaplan Meier estimates of overall survival in the intention-to-treat population of the metaanalysis on metronomic oral vinorelbine in non-small cell lung cancer.

Median (95% confidence interval [95%CI]) OS was 8.7 months (95%CI: 7.6–9.5). Overallsurvival rates at 6 months, one-year and at two years after starting metronomic oral vinorelbine were 64%, 30.3% and 8.9%, respectively (Fig 2). Successive suppression and reintroduction of the studies, one-by-one (jackknife) is shown in Table 3.

**Table 3:** Overall survival with successive suppression and reintroduction of the studies, one-by-one (jackknife).

	N	Median (months)	95%CI	% censored
*Overvall survival in the ITT population*	*418*	*8*.*7*	*7*.*6–9*.*5*	*11*.*2*
OS without study by Banna	377	8.2	7.5–9.6	9.8
OS without study by Bilir	383	9	7.7–9.6	12.3
OS without study by Briasoulis	388	9	7.9–9.8	11.9
OS without study by Camérini	375	8	7.5–9.6	11.5
OS without study by Kontopodis	372	8.7	7.7–9.8	9
OS without study by Mencoboni	344	9	7.6–9.7	13.4
OS without study by Barlesi	409	9	7.9–9.7	11.5
OS without study by Pasini	328	9	7.9–10	8.8
OS without study by D’Ascanio	368	7.8	6.9–9	12.8

In the univariate survival analysis, the covariates related to a shorter OS, with p< 0.20, were as follows: non-adenocarcinoma histology; PS = 2; anemia of any grade; grade3-4 anemia; thrombocytopenia of any grade (Table 4). Survival analysis did not significantly differ when patients receiving metronomic oral vinorelbine as upfront regimen were compared with patients receiving this schedule after first line systemic therapy failure (8.0 months [95%CI: 7.4–9.8] and 9.5 months [95%CI: 7.7–10.4] respectively; p = 0.92). The OS did not significantly differ between studies (p = 0.17).

**Table 4:** Univariate overvall survival analysis in the intention-to-treat population of the metaanalysis on metronomic oral vinorelbine in NSCLC.

Variables	Categories	N	Median (months)	95% IC	log-rank
Study	Banna et al.	41	9	5.3–13.3	0.17
	Bilir et al.	35	7.9	5.9–11.8	
	Briasoulis et al.	30	5.7	3.8–9.4	
	Camerini et al.	43	9	7–12	
	Kontopodis et al.	46	7.6	5.4–10.1	
	Mencoboni et al.	74	8	6–10	
	Barlesi et al.	9	4.6	0.4–7.2	
	Pasini et al.	90	7.5	5.2–9.7	
	D’Ascanio et al.	50	11.2	10.4–12	
Age	< 75 year	219	8	6.7–9.4	0.58
	> = 75 year	198	9	7.9–10.4	
Gender	Male	331	8.2	7.5–9.4	0.72
	Female	87	9.4	6–11.7	
Histology	ADE	204	9.7	8–10.6	0.09
	Non-ADE	184	8	7–9	
Stage grouping	3	75	10	9–11.8	0.24
	4	334	8	7.4–9.4	
ECOG performance status	0/1	238	9.8	8.1–10.5	<0.01
	2	180	7.1	5.8–9	
Vinorelbine dose	20/30	174	7.8	6.2–9.3	0.49
	40	53	10.6	9.9–11.7	
	50	182	9	7.5–10	
Vinorelbine chemotherapy sequence	Firstline	200	8	7.4–9.8	0.92
	2^nd^or subsequent	187	9.5	7.7–10.4	
Grade 3–4 neutropenia	Yes	33	5.7	3.8–8.2	0.29
	No	268	9.1	7.7–10	
Anémia of any grade	Yes	107	6.2	5.2–7.5	<0.01
	No	192	9.8	8–10.9	
Grade 3–4 anemia	Yes	10	6.2	3–9.3	0.02
	No	333	9	7.8–9.8	
Thrombocytopenia of any grade	Yes	10	8.9	0.7–13.6	0.12
	No	290	8.2	7.4–9.8	
Grade 3–4 thrombocytopenia	Yes	3	8.1	7.5–12.2	0.75
	No	298	8.5	7.4–9.8	
Nausea and vomiting of any grade	Yes	54	9.5	7.2–11.8	0.95
	No	246	8	7.1–9.7	
Grade 3–4 nausea or vomiting	Yes	12	9.9	3.5–12.7	0.56
	No	363	8	7.4–9.6	

**Fig 3:**
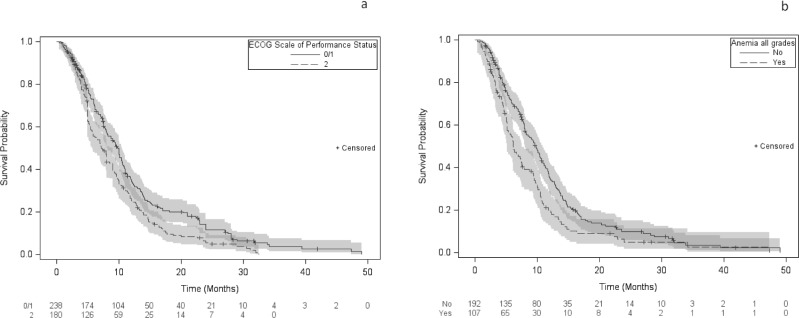
Kaplan Meier estimates of overall survival and adjusted hazard ratios in the intention-treat population of the metaanalysis on metronomic oral vinorelbine according to (a) performance status, (b) anemia of any grade.

Results of the Cox model stratified for the variable “study” are shown in Fig 3A and 3B: the following features were determinants of a poorer OS: ECOG PS = 2: adjusted hazard ratio (_adjusted_HR): 1.7 (95%CI: 1.21–2.39; p < 0.01) and anemia of any grade: _adjusted_HR: 1.44 (95%CI: 1.07–1.9; p = 0.02). As anemia was not specifically reported in the studies by Camerini et al and-Mencoboni et al., OS of patients in these two studies (n = 117) was compared with the remaining cohort (301). Median OS did not significantly differ (9.0 months [95%CI: 7.0–10.0] and 8.2 months [95%CI: 7.5–9.8], respectively; Log-rank p = 0.53) allowing reasonable generalization of the Cox model results to the intention-to-treat population.

### Progression-free survival

Median follow-up (range) for PFS analysis was 4.0 months (0.4–47.3). There were 327 events reported by the investigators in the nine studies (78.6% of the intention-to treat population). After penalization of two studies, (see methods section), 398 events (progressions) were considered in the PFS analysis (95.2%). Consequently, 4.8% of the patients were censored at the time of analysis.

**Fig 4:**
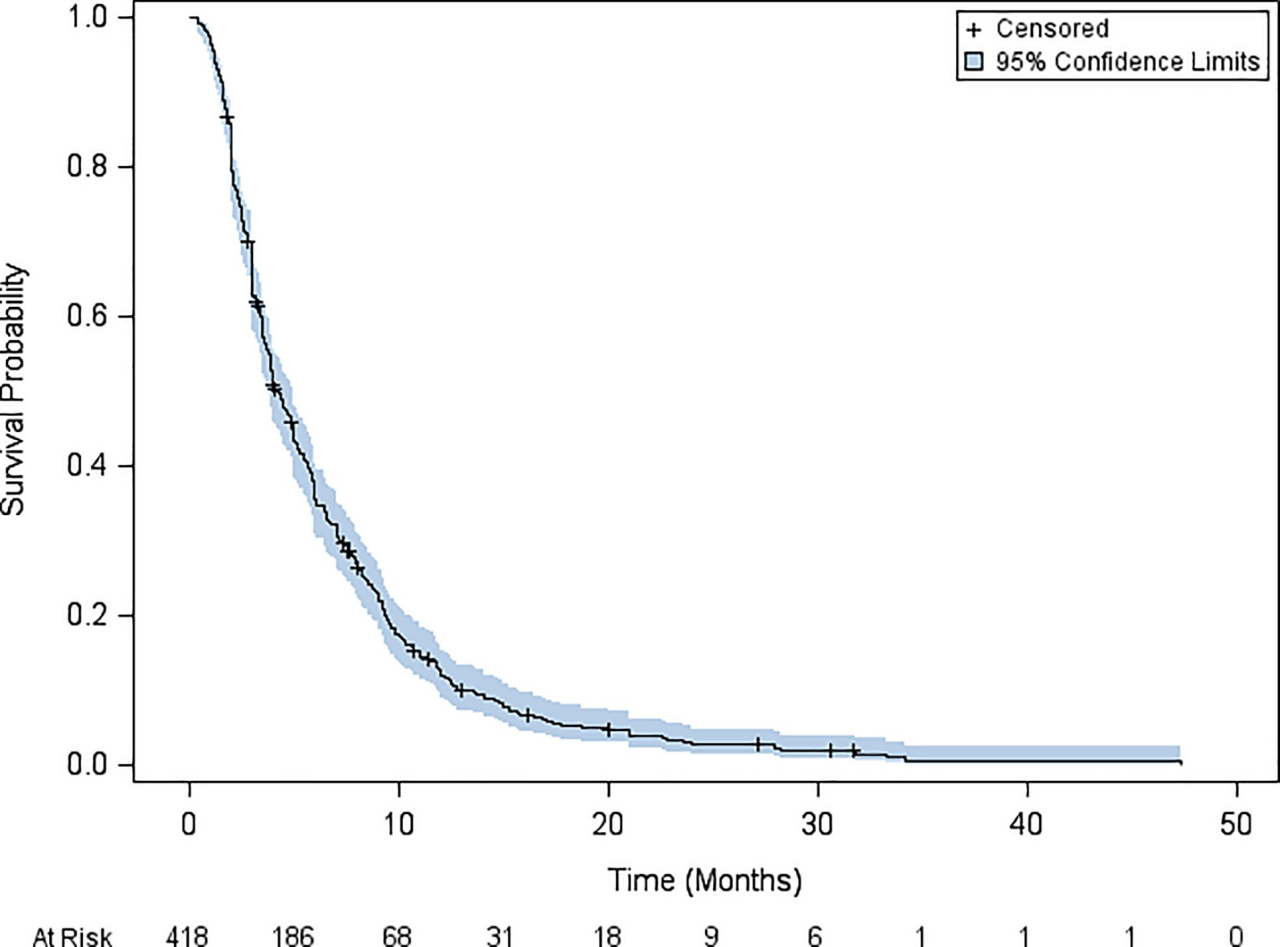
Kaplan Meier estimates of progression-free survival in the intention-to-treat population of the metaanalysis on metronomic oral vinorelbine in non-small cell lung cancer.

Median PFS was 4.2 months (95% CI: 3.9–5). Progression-free survival rates at 6 months and at one year after starting metronomic oral vinorelbine, were 35.0% and 11.9%, respectively (Fig 4). Successive suppression and reintroduction of the studies, one-by-one (jackknife) is shown in Table 5.

**Table 5:** Progression-free survival with successive suppression and reintroduction of the studies, one-by-one (jackknife).

	N	median (months)	95%CI	% censored
PFS in the entire ITT population	418	4.2	3.9–5	4.8
PFS without study by Banna	377	4.6	3.9–5.2	4
PFS without study by Bilir	383	4	3.6–5	5.2
PFS without study by Briasoulis	388	4.4	3.9–5	5.1
PFS without study by Camérini	375	4.2	3.7–5	4.8
PFS without study by Kontopodis	372	4.8	4–5.5	4.3
PFS without study by Mencoboni	344	4.4	3.9–5.2	5.8
PFS without study by Barlesi	409	4.4	3.9–5	4.9
PFS without study by Pasini	328	4.2	3.8–5	3.3
PFS without study by D’Ascanio	368	3.8	3.4–4	5.4

In the univariate analysis, the covariates related to a shorter PFS, with a p<0.20, were as follows: non-adenocarcinoma histology; ECOG PS = 2; stage grouping; anemia of any grade; grade 3–4 neutropenia and vinorelbine dose with a slightly detrimental effect of the highest dose (Table 6). In addition, the PFS differed among the studies (p < 0.01) due to the observed longer survival in the study by D’Ascanio et al.

**Table 6:** Univariate progression-free survival analysis in the intention-to-treat population of the metaanalysis on metronomic oral vinorelbine in NSCLC.

Variable	Categories	N	Median (months)	95%CI	log-rank
Study	Banna	41	3.2	2.5–3.8	<0.0001
	Bilir	35	5.9	3.9–9.8	
	Briasoulis	30	3.5	2.1–5	
	Camerini	43	5	3.5–6.5	
	Kontopodis	46	2.1	1.7–2.6	
	Mencoboni	74	3.7	3–5	
	Barlesi	9	1.7	0.4–3.9	
	Pasini	90	4.5	3.4–5.7	
	D’Ascanio	50	9.2	7.5–9.6	
Age	< 75 ans	219	3.9	3.5–5	0.57
	> = 75 ans	198	4.9	3.9–5.5	
Gender	Male	331	4	3.6–4.9	0.57
	Female	87	5	3.7–6.4	
Histology	ADE	204	4.8	3.9–5.9	0.16
	Non-ADE	184	4	3.5–5	
Stage grouping	III	75	6.4	4.5–8.2	0.034
	IV	334	4	3.5–4.9	
PS	0/1	238	5	3.9–5.5	0.037
	2	180	3.9	3.4–4.5	
Vinorelbine dose	20/30	174	4.1	3.6–5.1	0.018
	40	53	7.7	6.1–9.2	
	50	182	3.5	3–4.2	
Vinorelbine chemotherapy sequence	First line	200	4.7	3.9–5.5	0.77
	2nd or subsequent	187	4	3.4–5.2	
Grade 3–4 neutropenia	Yes	33	3.5	2–5.7	0.08
	No	268	4.4	3.9–5.3	
Anemia of any grade	Yes	107	3.8	2.8–4.4	0.077
	No	192	4.9	3.9–6	
Grade 3–4 anemia	Yes	10	3.8	1.2–5.5	0.16
	No	333	4.4	3.9–5.2	
Thrombocytopenia of any grade	Yes	10	5.5	0.7–10.2	0.52
	No	290	4.2	3.7–5.1	
Grade 3–4 thrombocytopenia	Yes	3	6	1–10.2	0.85
	No	298	4.2	3.7–5.1	
Nausea and vomiting of any grade	Yes	54	4.8	3.3–7.3	0.82
	No	246	4.2	3.7–5.1	
Grade 3–4 nausea and vomiting	Yes	12	7	1.2–9.8	0.80
	No	363	4.1	3.7–4.9	

In the Cox model stratified for the variables “dose” and “study”, a PS = 2 (HR: 1.61 [95%CI: 1.25–2.06]; p<0.01) and a stage IV disease (HR: 1.39 [95%CI: 1.03–1.88]; p = 0.03) were prognostic determinants of a shorter PFS.

## Safety

Overall, toxicity was mild to moderate and manageable. Forty percent of the patients experienced no toxicity at all. Among the 247 remaining patients (59.1%) of the cohort, 66 (15.8%) experienced a grade 3–4 toxicity. The most frequent toxicity was anemia experienced by35.79% of the patients. Only 10 patients (2.9%) have had a grade 3–4 anemia. Grade 3–4 neutropenia affected around 11.0% of the cohort. Nausea and vomiting affected 18.0% of the cohort with 12patients (3.2%) experiencing a grade 3–4 emesis (Table 7).

**Table 7:** Percentage of patients affected by toxicity in the intention-to-treat population of the metaanalysis on metronomic oral vinorelbine in NSCLC.

Descriptive statistics	%	9%CI
*Overall toxicity of any grade*	*59*,*1*	*54*.*2–63*.*8*
*Overall toxicity of grade 3–4*	*15*,*8*	*12*.*5–19*.*7*
Neutropenia of any grade	23.0	18.4 28.3
Grade 3–4 neutropenia	10.9	7.8–15.2
Anemia of any grade	35.8	30.4–41.54
Grade 3–4 anemia	2.9	1.5–5.5
Thrombocytopenia of any grade	3.3	1.7–6.24
Grade 3–4 thrombocytopenia	1.0	0.3–3.1
Nausea and vomiting of any grade	18.0	13.9–22.9
Grade 3–4 nausea and vomiting	3.2	1.7–5.7

Toxicity affected more patients in the subgroup of the cohort that received a 50 mg oral metronomic dose of vinorelbine in comparison with the 30–40 mg vinorelbine doses. This difference was statistically significant for the following features of toxicity: Overall toxicity of any grade (50.7% versus 67.6% for the 30–40 mg and 50 mg doses, respectively; p <0.01); grade 3–4 neutropenia (6.6% versus 21.5% respectively; p <0.01 0.0003); and anemia of any grade (29.3% versus 50.8%; p <0.01).

## Discussion

This metaanalysis included individual patient-data from nine phase 2 single arm studies, encompassing 418 patients with advanced NSCLC patients. Most patients presented with unfavorable prognostic features such as older age, stage IV disease, high comorbidity score, ECOG PS = 2, or third line or subsequent line of systemic therapy. In this cohort, metronomic administration of oral vinorelbine had a good safety profile with mild to moderate hematologic and digestive toxicity albeit the highest dosage (50 mg) induced a slightly higher toxicity rates. Median OS, one year and two year survival rates were 8.7months, 30.3% and 8.9% respectively. Median PFS, six months and one year PFS rates were 4.2 months, 35% and 11.9% respectively. Multivariate analysis showed that an ECOG PS = 2, and occurrence of anemia of any grade, were prognostic determinants of poor OS, whereas neither vinorelbine dose nor “study effect” variable significantly influenced survival. As the 50mg vinorelbine dose induced higher rates of anemia. As the outcome of patients did not significantly vary according to doses, 30–40 mg thrice weekly appeared to have the optimal risk/benefit ratio.

In the cohort herein, survival of patients receiving vinorelbine as single-drug regimen compared well with current knowledge regarding the outcome of patients receiving more conventional chemotherapy for stage IV NSCLC. For instance, in a recent epidemiological study of the national cancer data base (NCDB), the authors investigated the two-year survival from date of diagnosis to death in 193,279 patients with metastatic NSCLC [19]. Two-year survival improved from 9.9% in 1998 to 14.6% in 2010. The improvement was mainly observed for adenocarcinoma and was thought to be driven by the emergence of targeted therapies for these patients suffering from an adenocarcinoma with actionable mutations. The two-year survival rate of 8.9% of our cohort is almost similar to the 1998 two-year survival of the NCDB but differs from the more recent value. The proportion of adenocarcinoma in the NCDB cohort and in our intention-to-treat population was similar (47.8% and 48.8% respectively) but the definition of survival was different. In the NCDB, survival was calculated from the date of diagnosis, whereas in our metaanalysis, survival was calculated from the first day of metronomic oral vinorelbine until the date of death of any cause. As up to 44.7% of the patients received vinorelbine as a second-line or subsequent-line systemic therapy, our survival analysis, unlike that described in the NCDB study, did not record the entire disease course. In addition, many of the patients suffering from adenocarcinoma with actionable mutations may have probably received tyrosine kinase inhibitors during previous sequences of treatment. This would cancel out the survival advantage of targeted therapy.

Another comparison might be attempted with recent studies in pretreated metastatic NSCLC patients. Two separate studies have compared nivolumab and docetaxel in patients who had progressed after platinum-based doublet chemotherapy. In the study conducted in non-squamous NSCLC [20], one-year-survival with docetaxel was 39%, whereas the study of squamous-cell cancer [21],it was 24%. In our cohort the one-year survival rate reached 30.3%. The OS of our intention-to-treat population should be interpreted account the frailty of most of the patients. Although, 80.9% of the patients had at least one poor prognostic feature such has an ECOG PS = 2 or a Charlson comorbidity score > 2, the one-year survival compared well with the docetaxel group of the nivolumab studies for which these frailty characteristics were an exclusion criterion.

The safety profile of the metronomic oral vinorelbine appears to be better than that of its conventional regimen (intravenous route and 30 mg/m^2^ dosage given on day 1 and 8 of a three-week cycle). For instance, in the monotherapy vinorelbine group of the study by Le Chevalier et al [22], which compared three different regimens, 53.2% of the patients experienced a grade 3–4 neutropenia, versus 11% in the metaanalysis herein. A similar comment might be made regarding nausea and vomiting that are important aspects of safety profile, they are among the most feared adverse events. The nine studies encompassed in the metaanalysis had broader eligibility criteria and allowed patients with unfavorable covariables to receive an active treatment. Therefore, the intention-to-treat population of the herein metaanalysis, has demographic and disease characteristics usually observed in the real world practices.

There are limitations of our work. One can point out that the original studies had critical limitations due to small sample size. With a median contribution of each study to the metaanalysis by 43 patients, the sample size of most of the studies, belongs to a conventional single-arm phase 2 population. Morevover, individual-patient data metaanalyses are the optimal tools to circumvent the limited size of individual phase 2 trials. Secondly, the quality of the PFS evaluation in a metaanalysis could be questioned. This is a known limit of metaanalyses, aggregating studies with different follow-up schedules. Nevertheless, PFS in NSCLC is always evaluated at short intervals of time (no more than 2 months for patients with active treatment and no more than 3 months for patients during post-study follow-up). Moreover, the overall survival, that is not a soft endpoint as PFS is, showed reliable activity of metronomic oral vinorelbine. One can suggest that randomized phase 2 studies would have been an alternative to single-arm phase 2 studies. As a matter of fact, non-comparative phase 2 design is a good alternative to classic single-group phase 2 trial designs for evaluating an experimental agent. The reason is that single arm phase 2 studies are subjects of multiple putative biases such as their inability to separate treatment effect of a given drug (or a given regimen) from trial effects (such as patient selection, eligibility, and schedule of efficacy assessment). Nevertheless, randomized phase 2 trials are non-comparitive by essence and do not allow outcome comparison between patients treated with the investigational schedule and these receiving the standard treatment. Although a metaanalysis of individual data from 418 patients allows for an accurate appraisal of the survival, it cannot replace a head-to-head comparison with a standard regimen. However, this individual-patient data metaanalysis gives reliable information able to design hypothesis for further comparative phase 3 studies. The good safety profile is supposed to preserve the quality of life of frail patients, such as elderly subjects or patients suffering from comorbid conditions. Quality of life is an important consideration in treatment choice. Unfortunately, there was no quality of life assessment in the original studies (a good safety profile, abeilt one of the conditions for a preserved quality of life, is not a surrogate). In two of the nine studies, the lack of sufficiently updated survival data may have had artificially inflated the survival rate. In order to compensate for this phenomenon (informative censorship), a penalized evaluation of survival was applied. This method ascertains that survival data are not overestimated by insufficient updating of follow-up. In the present study, it was conservative to consider censorship as informative, considering the high rate of death in metastatic lung cancer.

Survival and safety results presented herein suggest that further evaluation of metronomic oral vinorelbine is warranted. In previously treated patients, the comparison of this regimen with the standard docetaxel single-drug regimen might be of interest. Endpoints such as quality of life should also be evaluated aside conventional survival analyses. The effects of a combination of metronomic oral vinorelbine with anti-PD-1 antibody might also be considered. Finally, mathematical models might help search of optimal doses.

In conclusion, metronomic oral vinorelbine is an active and well-tolerated single-drug chemotherapy regimen in metastatic NSCLC and is a manageable therapy in frail patients.

## Supporting information

S1 File Data_ metronomic oral vinorelbine. (XLS)

S2 File PRISMA-2009-Checklist-MS-Word. (DOC)
